# Relation between lipogranuloma formation and fibrosis, and the origin of brown pigments in lipogranuloma of the canine liver

**DOI:** 10.1186/1476-5926-7-5

**Published:** 2008-05-12

**Authors:** Kaori Isobe, Hiroyuki Nakayama, Koji Uetsuka

**Affiliations:** 1 Department of Veterinary Pathology, Graduate School of Agricultural and Life Sciences, University of Tokyo, Japan

## Abstract

**Background:**

In a previous study we confirmed that canine hepatic lipogranuloma, defined as lesions consisting of small round cells which contain lipid vacuoles and brown pigments in their cytoplasm, was an assembly of Kupffer cells and/or macrophages, and that the cytoplasmic brown pigments in the lesions were hemosiderin and ceroid. However, the pathogenesis of the lesion remains unclear. Kupffer cells (resident macrophages) play a key role in hepatic fibrogenesis due to the production of cytokines including TGF-β. In the present study, we have examined 52 canine liver samples (age: newborn – 14 years; 25 males and 27 females) and investigated the correlation between lipogranuloma formation and fibrosis as well as the origin of brown pigments of lipogranulomas.

**Results:**

Lipogranulomas were detected histopathologically in 23 (44.2%) of the 52 liver samples. No significant correlation was found between the density of lipogranulomas and distribution of collagen type I/III in the liver. Pigmentation of lipogranulomas showed significant correlations with that on both hepatocytes and sinusoidal cells, indicating that pigments of lipogranuloma (hemosiderin and ceroid) might be derived from hepatocytes and Kupffer cells.

**Conclusion:**

Lipogranulomas are not a contributing factor in hepatic fibrosis, but might be a potential indicator of the accumulation of iron and lipid inside the liver.

## Background

Lipogranulomas, also termed fatty cysts, are often found in the hepatic parenchyma of dogs [1,2], especially of those with portosystemic shunt (PSS) [3-6], and are defined as lesions consisting of small round cells which contain lipid vacuoles and brown pigments in their cytoplasm, although the amounts of vacuoles and pigments vary among lesions. Besides the canine liver, lipogranulomas are observed in the rat as well as in human livers with cirrhosis [7-10], and are considered to be involved in hepatic cirrhosis in human medicine [9,10]. However, in canine cases, the significance of lipogranulomas in the pathogenesis of cirrhosis is still not clear.

Our previous study [11] confirmed that hepatic lipogranuloma consisted of Kupffer cells and/or macrophages, and the cytoplasmic brown pigments were hemosiderin and ceroid, although the pathogenesis of canine lipogranuloma remains unclear. Kupffer cells (resident macrophages) play a key role in hepatic fibrogenesis due to the production of cytokines including transforming growth factor-β (TGF-β) [12-14]. TGF-β, one of the most pro-fibrotic cytokines, is necessary and sufficient for the induction and progression of fibrotic lesions, and may serve as the initiating event in the activation of myofibroblasts, which then secrete a large amount of extracellular matrix [15]. We therefore supposed that the component cells of lipogranulomas might have the potential to cause hepatic fibrosis.

Moreover, it is uncertain from where the pigments of canine lipogranuloma are derived. Although it is already demonstrated that hepatocytes are not directly involved in lipogranuloma formation [11], it might be possible that hepatocytes containing pigments are phagocytized by Kupffer cells, which then form a lipogranuloma.

In the present study, we histopathologically examined the correlation between lipogranulomas and fibrosis, and speculated the origin of brown pigments of lipogranulomas.

## Results

### Histopathology of the liver

Histopathological diagnoses of the 52 canine autopsy cases examined in the present study are shown in Table 1. Histopathological changes in the liver were observed in most cases. In cases 8, 19, 28, 34, 36 and 38, tumor metastases to the liver were observed. In case 18, thrombi were formed in large vessels in the portal area. In case 52, nodular proliferation of well-differentiated hepatocarcinoma was focally observed. In case 41, cholangiocarcinoma proliferated densely with a tubuliform pattern.

**Table 1:** Canine autopsy cases examined.

**Case No.**	**Sex**^a)^	**Age**^b)^	**Breed**	**Lipogranulomas**	**Main diagnosis**
1	M	0 y	Labrador Retriever	-	Systemic hyperemia/congestion
2	M	0 y	Labrador Retriever	-	Systemic hyperemia/congestion
3	F	7 m	Mongrel	+	Renal dysplasia
4	Mc	3 y 1 m	Chihuahua	-	Necrotizing meningoencephalitis
5	F	3 y 5 m	Shetland Sheepdog	-	Thymoma, Septicemia
6	M	3 y 10 m	Shibainu	-	Severe enteritis, Nephritis
7	F	4 y	Miniature Dachshund	-	Malignant lymphoma
8	M	4 y	Bernese Mountain Dog	-	Malignant lymphoma
9	Mc	4 y 4 m	American Cocker Spaniel	-	Meningoencephalitis
10	Fs	4 y 5 m	Maltese	+	Necrotizing meningoencephalitis
11	M	4 y 6 m	Miniature Dachshund	-	Thrombocytopenia
12	Fs	5 y 2 m	Miniature Schnauzer	-	Chronic interstitial nephritis
13	Mc	5 y 4 m	Miniature Dachshund	-	Gastroduodenitis
14	F	6 y	Miniature Dachshund	+	Malignant melanoma
15	F	6 y 2 m	Shih Tzu	-	hepatic and renal calcinosis
16	Mc	6 y 3 m	Akitainu	-	Peritonitis, Septicemia
17	F	7 y	Labrador Retriever	+	Chronic myelocytic leukemia
18	M	7 y 4 m	English Cocker Spaniel	+	Thrombosis
19	Mc	7 y 4 m	Golden Retriever	-	Malignant lymphoma
20	F	7 y 9 m	Shetland Sheepdog	-	Chronic bronchopneumonia
21	F	8 y 1 m	Shih Tzu	-	Enteritis, Interstitial nephritis
22	F	8 y 3 m	Pug	+	Necrotizing meningoencephalitis
23	M	8 y 9 m	Yorkshire Terrier	+	Catarrhal pneumonia
24	Fs	9 y	Golden Retriever	-	Mammary carcinoma
25	M	9 y	Miniature Pinscher	+	Malignant mesothelioma
26	M	9 y 3 m	Labrador Retriever	+	Malignant lymphoma
27	M	9 y 6 m	Miniature Schnauzer	+	Hemangiosarcoma
28	M	9 y 7 m	Whippet	-	Malignant histiocytoma
29	F	9 y 9 m	Golden Retriever	+	Malignant mesothelioma
30	M	9 y 9 m	Golden Retriever	-	Encephalatrophy
31	M	9 y 9 m	Welsh Corgi Pembroke	-	Pulmonary calcinosis
32	M	10 y	German Shepherd Dog	-	Infarction of heart, kidney, lung
33	F	10 y 4 m	Shih Tzu	-	Intestinal hemorrhage
34	F	10 y 7 m	Welsh Corgi Pembroke	-	Malignant lymphoma
35	Mc	10 y 11 m	Labrador Retriever	+	Acute leukemia
36	Fs	11 y	Mongrel	-	Fibrosarcoma
37	F	11 y 3 m	Shih Tzu	-	Acute lymphocytic leukemia
38	F	11 y 3 m	Labrador Retriever	+	Mammary carcinoma
39	M	11 y 5 m	Beagle	-	Malignant lymphoma
40	M	11 y 5 m	Shetland Sheepdog	-	Malignant histiocytoma
41	Fs	11 y 5 m	Cavalier King Charles Spaniel	-	Uremic pneumonia
42	F	11 y 10 m	Miniature Dachshund	+	Cholangiosarcoma
43	Mc	12 y	Golden Retriever	+	Hemangiosarcoma
44	F	12 y 5 m	Shih Tzu	-	Chronic interstitial nephritis
45	Fs	12 y 6 m	Mongrel	+	Malignant lymphoma
46	F	13 y	Miniature Dachshund	+	Fibrinopurulent pneumonia
47	Fs	13 y	Mongrel	+	Transitinal carcinoma
48	F	13 y 9 m	Long Coat Chihuahua	+	Chronic nephritis
49	F	13 y 9 m	Miniature Dachshund	+	Malignant lymphoma
50	M	14 y	Shih Tzu	+	Gastric perforation
51	Fs	14 y	Labrador Retriever	+	Cardiac calcinosis, Thrombosis
52	Mc	14 y 3 m	Italian Greyhound	+	Bronchial adenocarcinoma

a) M: male; Mc: male castrated; F: female; Fs: female spayed. b) y: years; m: months.

### Incidence and density of lipogranulomas

Among 52 autopsy cases, lipogranulomas were found in the livers of 23 cases (44.2%) (Table 1). The density of lipogranulomas was classified into scores 0 to 3: score 0: 29 cases (55.8%); score 1: 14 cases (27.0%); score 2: 6 cases (11.5%); and score 3: 3 cases (5.8%) of total 52 canine cases. The mean score was 1.5 ± 0.7 (mean ± S.D.).

### Fibrosis score

Fibrosis was graded with scores 0 to 3 (Table 2) according to the amount and distribution of collagen. The mean scores for fibrosis were: collagen type I; 1.6 ± 1.1, and type III; 2.3 ± 0.6. Neither score was statistically related to that of lipogranuloma density.

**Table 2:** Scoring criteria for distribution of collagen type I/III, and pigmentation in the liver.

**Score**	**Distribution of collagen type I/III**	**Pigmentation**
0	None	None
1	Thin collagen fibers are occasionally observed in the foci of hepatocytic changes and/or periportal area	Light
2	Distinct collagen fibers are observed in the foci of hepatocytic changes and/or periportal area	Moderate
3	Thick and distinct collagen fibers are observed in the foci of hepatocytic changes and periportal area	Severe

### Pigmentation score

**Figure 1:**
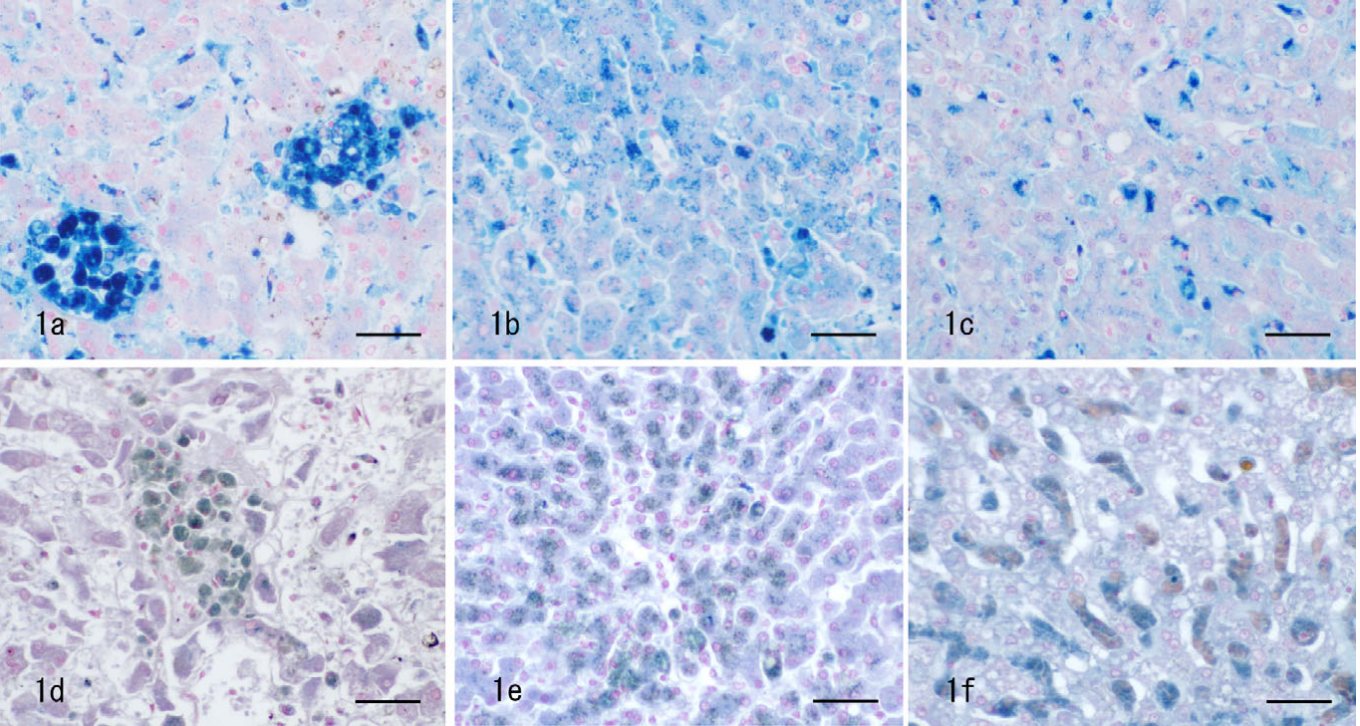
**Pigmentation in lipogranulomas, hepatocytes and sinusoidal cells**. Brown pigments are positive for Berlin blue inside lipogranulomas (a), hepatocytes (b) and sinusoidal cells (c), and for Schmorl in lipogranulomas (d), hepatocytes (e) and sinusoidal cells (f). Bar = 20 μm.

Lipogranuloma is defined as an aggregation of cells containing lipid vacuoles and brown pigments in the cytoplasm, as mentioned above. The pigments were positively stained with Berlin blue and Schmorl (Fig. 1a,d). Such pigments were seen also in the cytoplasm of some hepatocytes (Fig. 1b,e) and sinusoidal cells (Fig. 1c,f). The scores of pigmentation are summarized in Table 3, indicating 2.2 ± 1.1 (Berlin blue) and 2.2 ± 0.7 (Schmorl) of lipogranuloma, 1.3 ± 0.9 (Berlin blue) and 2.2 ± 0.6 (Schmorl) of hepatocytes, and 2.3 ± 1.1 (Berlin blue) and 2.1 ± 0.8 (Schmorl) of sinusoidal cells. Regarding the amount of Berlin blue-positive iron pigments in lipogranulomas, hepatocytes and sinusoidal cells, positive correlations were mutually found among them (P < 0.05) (Table 3). Schmorl-positive ceroid pigmentation in lipogranulomas was positively correlated both with that on hepatocytes and sinusoidal cells from the results of Schmorl (P < 0.05), although no correlation was observed between pigmentation in hepatocytes and sinusoidal cells (Table 3).

**Table 3:** Estimated scores of pigmentation of lipogranulomas, hepatocytes and sinusoidal cells.

	**Berlin blue**	**Schmorl**
Lipogranulomas	2.2 ± 1.1^a^	2.2 ± 0.7^a^
Hepatocytes	1.3 ± 0.9^b^	2.1 ± 0.6^c^
Sinusoidal cells	2.3 ± 1.1^c^	2.1 ± 0.8^c^

Score = Mean ± S.D. Within a column, values with different superscript letters differ (P < 0.05).

## Discussion

Hepatic lipogranulomas are defined as lesions consisting of small round cells which contain lipid vacuoles and brown pigments in their cytoplasm, although the amounts of vacuoles and pigments vary among lesions. Our previous study [11] confirmed that the lesions consisted of Kupffer cells and/or macrophages, and the cytoplasmic brown pigments are hemosiderin and ceroid.

Macrophages play a very prominent role in fibrotic diseases [15]. Resident and/or infiltrating macrophages play a critical part in initiation of myofibroblast conversion from precursor fibroblasts, fat-storing cells (Ito cells), and endothelial cells [15]. Among them, Kupffer cells (resident macrophages) play a key role in fibrogenesis due to the production of transforming growth factor-β (TGF-β) [12-14], hepatocyte growth factor (HGF) [16], tumor-necrosis factor-α (TNF-α) and nitric oxide, which in turn activate fat-storing cells [17-19].

As no significant correlation was found between the density of lipogranulomas and distribution of collagen fibres, lipogranuloma may not be directly involved in fibrogenesis of the liver. Since hepatic fibrosis is a complex process that involves many hepatic cells other than Kupffer cells and/or macrophages, it might be possible that the phagocytes could just trigger initiation of fibrotic events, but could not amplify the fibrotic response which is necessary for hepatic fibrosis.

**Figure 2:**
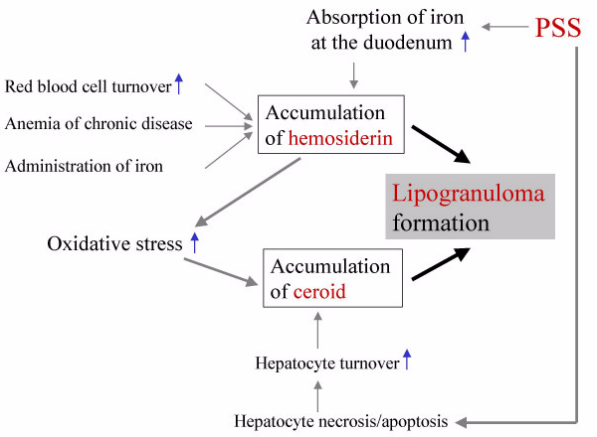
Mechanism diagram of lipogranuloma formation.

The abnormal metabolism of iron and lipid may cause the accumulation of hemosiderin and ceroid, respectively. Accumulation of hemosiderin may indicate an increase of red blood cell turnover, and that of ceroid may be a result of increased hepatocyte turnover [2]. Iron and ceroid accumulation is involved in increased oxidative stress with iron-catalyzed production of reactive oxygen species causing oxidative damage to lipids, proteins, and other molecules [20,21]. This mechanism may bring about the accumulation of iron and ceroid in hepatocytes, which are then phagocytized by Kupffer cells or macrophages, and subsequently a closely-aggregated "lipogranulomas" are formed (Fig. 2). In canine PSS cases, on the other hand, increased iron absorption at the duodenum [22] and increased accumulation of ceroid caused by abnormal lipid metabolism in the hepatocytes, might be key factors in forming lipogranulomas (Fig. 2). Since hemosiderin and ceroid are end metabolic products of iron and lipid, respectively, the pigments remain in phagocytes once they are phagocytized [21,23].

As demonstrated in our previous study [11], hepatocytes are not mainly involved in the formation of lipogranulomas. Here, we can propose two hypotheses regarding the mechanism of lipogranuloma formation. One is that the vacuolated hepatocytes with brown pigments (or iron/lipid) in their cytoplasm are phagocytized by Kupffer cells, and the other is that free pigments (or free iron/lipid in blood) are directly phagocytized by Kupffer cells. Since the pigmentation score of lipogranulomas showed positive correlation with that of both hepatocytes and sinusoidal cells, both hypotheses were thought to be possible.

Given the above, we considered that the pigments of lipogranulomas could be derived from both hepatocytes and Kupffer cells. Moreover, lipogranulomas might be a potential indicator of accumulation of iron and lipid inside the liver.

## Conclusion

There was no correlation between the density of lipogranulomas and the distribution of fibrosis in the canine liver. Pigmentation of hemosiderin and ceroid in lipogranulomas had significant correlations with that in hepatocytes and in sinusoidal cells, respectively, indicating that these pigments in lipogranuloma might be derived from both hepatocytes and Kupffer cells. It is concluded that lipogranulomas are not a contributing factor for hepatic fibrosis, but a potential indicator for the accumulation of iron and lipid inside the liver.

## Methods

### Liver samples

The liver samples used in the present study were obtained from 52 dogs autopsied between January 2005 and December 2006 at the Department of Veterinary Pathology, the University of Tokyo. The dogs comprised 25 males and 27 females ranging from newborn to 14 years old (Table 1).

### Histopathological methods

Excised liver tissues were fixed in 10% neutral-buffered formalin, embedded in paraffin, and sectioned at 4 μm. The paraffin sections were stained with hematoxylin and eosin (HE). For further histopathological examination, Berlin blue stain, Schmorl reaction, and immunostains were performed.

For immunostain, deparaffinized sections were autoclaved or digested in 1% trypsin for antigen retrieval, and then immersed in 0.3% hydrogen peroxidase to block internal peroxidase activity, and in 8% skimmed milk to block non-specific binding of the primary antibody. The primary antibodies used were: anti-rat collagen type I, rabbit serum (LSL CO., Cosmo Bio, Tokyo, Japan), diluted 1:400; and anti-mouse collagen type III, rabbit serum (LSL CO., Cosmo Bio), diluted 1:200. The sections were then reacted with each biotinylated secondary antibody (KPL, Gaithersburg, MD, U.S.A.), incubated with peroxidase-labeled streptavidin (Dako, Glostrup, Denmark), and visualized with 3,3'-diaminobenzidine-tetrahydrochloride (DAB) as chromogen. Counterstaining was done with methyl green.

### Examination of liver sections

Fibrosis in the liver was evaluated using anti-rat collagen type I and anti-mouse collagen type III antibodies. Pigmentation on lipogranulomas, hepatocytes and sinusoidal cells was also evaluated using Berlin blue-stain or the Schmorl reaction sections. Lipogranuloma density was herein determined by counting the number of lipogranulomas in 5 images (3,200 × 2,560 pixels), taken from each HE section with a ×10 objective lens. The total number of lipogranulomas in the 5 images was considered the lipogranuloma density per a defined area unit. The density of lipogranulomas was classified into 4 scores, 0 to 3; score 0: total number of lipogranulomas was zero; score 1: below ten; score 2: below twenty; and score 3: twenty-one and above.

Scoring criteria to determine the distribution of collagen type I/III in the liver are shown in Table 2. Accumulation of brown pigments was assessed by classifying the amount of pigments in lipogranulomas, hepatocytes and sinusoidal cells. The scoring criteria of pigmentation are also shown in Table 2.

### Statistical methods

Spearman rank correlation coefficients were used to test the association between the density of lipogranuloma and distribution of collagen type I/III, and to investigate the origin of brown pigments of lipogranulomas [24]. P values less than 0.05 were considered to indicate significant differences.

## Competing interests

The authors declare that they have no competing interests.

## Authors' contributions

KI performed most of the experiments and prepared the manuscript. HN and KU provided assistance for the preparation of the manuscript. KU participated in the design of the study. All authors have read and approved the content of the manuscript.
